# Special Issue of the Manufacturing Engineering Society (MES)

**DOI:** 10.3390/ma11112149

**Published:** 2018-10-31

**Authors:** Eva María Rubio, Ana María Camacho

**Affiliations:** Department of Manufacturing Engineering, Industrial Engineering School, Universidad Nacional de Educación a Distancia (UNED), St/Juan del Rosal 12, E28040 Madrid, Spain; amcamacho@ind.uned.es

**Keywords:** additive manufacturing and 3D printing, advances and innovations in manufacturing processes, sustainable and green manufacturing, manufacturing systems: machines, equipment and tooling, metrology and quality in manufacturing, Industry 4.0, product lifecycle management (PLM) technologies, production planning, risks

## Abstract

Research in the field of materials is very broad, ranging from studies on the structure and properties at the atomic or molecular level to the most complex or sophisticated applications that can be done with them, as well as studies about other aspects related to their processing, use or management. The Special Issue of the Manufacturing Engineering Society (MES), published in the Section “Manufacturing Processes and Systems” of the journal *Materials*, focuses, mainly, on the applications and key processing aspects of materials, collecting a set of 48 original papers focused on the field of manufacturing engineering and materials processing.

The Special Issue of the Manufacturing Engineering Society (MES), published in the Section “Manufacturing Processes and Systems” of the journal *Materials*, was born as an initiative from the Manufacturing Engineering Society of Spain, with the aim to spread outstanding works in which the latest cutting-edge advances in the field of manufacturing engineering and materials processing have been presented.

This Special Issue explored the evolution of traditional manufacturing models towards the new requirements of the Manufacturing Industry 4.0; publishing, finally, 48 papers (47 research papers and one concept paper) in the main topics covered by this Special Issue, concretely in: additive manufacturing and 3D printing; advances and innovations in manufacturing processes; manufacturing systems: machines, equipment and tooling, metrology and quality in manufacturing; product lifecycle management (PLM) technologies; and manufacturing engineering and society. 

Likewise, in some of them, without being the main or most outstanding subjects of the work, the following topics have been also addressed: sustainable and green manufacturing; manufacturing of new materials; manufacturing automation; design, modeling and simulation in manufacturing engineering; and production planning.

Contributions on emerging methods and technologies, such as those related to additive manufacturing and 3D printing [1,2,3,4,5,6,7,8,9,10,11,12,13,14], have been very numerous, which is not surprising as they are technologies with a lot of potential.

The number of contributions in the “advances and innovations in manufacturing processes” field has been very remarkable as well, where outstanding works have been presented in the following areas: machining [15,16,17,18,19,20,21,22,23,24], forming [25,26,27,28,29,30], moulding [31], welding [32,33], and non-traditional manufacturing processes [34,35,36,37,38].

No less important, as a whole, are the works on: manufacturing systems: machines, equipment and tooling [39,40,41,42], metrology and quality in manufacturing [43,44,45], product lifecycle management (PLM) technologies [46] and, risks, within the topic “manufacturing engineering and society” [47,48].

The Special Issue has been an excellent means of exposing some of the main current lines of research of members and collaborators of the MES, promoting the internationalization of their results and the presence of the MES itself in the international scientific-technical landscape, widely fulfilling the aims initially proposed.

It is remarkable how many readings and downloads of papers belonging to this Special Issue have been done, after only three months since the publication of the first work, showing the great interest that all these topics arouse in readers of the journal *Materials*; particularly topics related to the processing of materials.

## In Memoriam

We want this Special Issue to serve as a posthumous tribute to our colleague and friend Mariano Marcos Bárcena, who participated very actively in the genesis of this thrilling project, and whose contribution to manufacturing engineering is present in this Special Issue, both explicitly, in a concrete work, and implicitly, in several papers by his research group from the University of Cadiz who have kept working on the investigation lines promoted by him.
